# Deep Eutectic Solvents for Bast Fibre Degumming: A Cross-Crop Review of Systems and Mechanisms

**DOI:** 10.3390/molecules31213911

**Published:** 2026-10-29

**Authors:** Yuan Liu, Chunjie Liu, Haohan Zhao, Jikang Chen, Siqi Huang, Liangliang Liu

**Affiliations:** Institute of Bast Fiber Crops, Chinese Academy of Agricultural Sciences, Changsha 410205, China; liuyuan06@caas.cn (Y.L.);

**Keywords:** deep eutectic solvent, bast fibre, degumming, ramie, hemp, kenaf, flax, *Apocynum venetum*, solvent recycling

## Abstract

This review places deep eutectic solvent (DES) degumming of the five bast fibre crops—ramie, hemp, kenaf, flax and *Apocynum venetum*—on a common basis, covering systems, conditions, outcomes, mechanisms and reuse records. It draws on eighteen research studies and one dedicated review, prioritising quantified works indexed in the Science Citation Index. The field has converged on a narrow solvent family, with choline chloride the near-universal hydrogen bond acceptor and choline chloride–urea best on ramie and kenaf and most balanced on hemp. Processes run at 100–175 °C with microwave assistance shortening treatment time; residual gum falls to 3.15% on ramie and 9.42% on kenaf, with reuse demonstrated over three to five cycles. Bond-level mechanistic evidence, however, covers only hemp and kenaf, leaving the consensus mechanism inferred for most crops. No solvent mass balance has been reported, leaving the energy and water demand of regeneration, and hence the claimed environmental advantage, unquantified. Degumming performance is judged by multiple indicators, and without weighting schemes or standardised protocols the reported optimal conditions are not directly comparable. DES degumming of bast fibres is therefore technically verified but industrially unvalidated; process scaling and long-term solvent recycling are priorities for deployment.

## 1. Introduction

### 1.1. Industrial Role and Fibre Architecture of the Five Bast Fibre Crops

Ramie, hemp, kenaf, flax and *Apocynum venetum* (Luobuma in Chinese) are the five principal bast fibre crops cultivated for their long, strong and low-density cellulosic fibres. With a long-standing application in textiles, these fibres have also become recognised reinforcements for polymer composites over the past two decades, where their specific stiffness, damping behaviour and biodegradability compare favourably with those of glass fibre [1,2,3]. The five crops are grown on very different scales and for different markets, and it is this commercial position, instead of any botanical distinction, that decides how much industrial and research attention each fibre product receives. Ramie remains a textile fibre spun in East Asia for summer fabrics and traded on its lustre, moisture uptake and strength, and it has also been assessed with nettle as a reinforcement in polylactic acid biocomposites [4]. Flax is Europe’s established bast fibre crop and the origin of linen, and it is the most widely used bast reinforcement in polymer composites [2]. Hemp fibre has returned to textiles and to composites after decades of regulatory restriction [5]. Kenaf grows quickly on marginal land and supplies paper, board and packaging before textiles, with marine composites now added to that list [3]. *Apocynum venetum* occupies the smallest niche of the five. It tolerates saline and drought-prone soils, and is harvested both as a fibre and as a medicinal plant [6]. Its fibre has been characterised mainly through composition, structure and properties [7,8]. The separation of the fibre from the stem has been studied most thoroughly for the crops that carry a large textile or composite market [9,10], which is also where the strongest incentive for a cleaner separation route exists.

**Figure 1:**
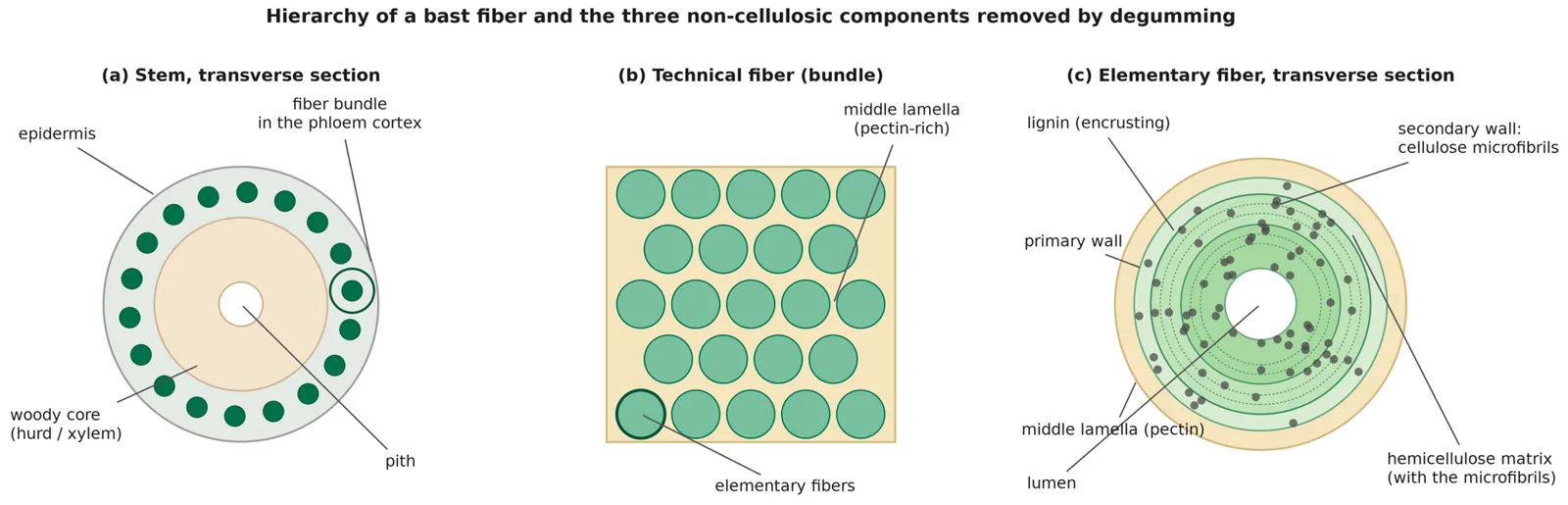
Hierarchy of a bast fibre, from stem to elementary fibre. (**a**) Transverse section of the stem: the fibre bundles sit in the phloem cortex, outside the woody core. (**b**) A technical fibre: elementary fibres cemented along their length by a middle lamella rich in pectin. (**c**) An elementary fibre in cross-section: a cellulose secondary wall (aggregated microfibrils) enclosing the lumen, encrusted with lignin and sheathed by a hemicellulose–pectin matrix. The three non-cellulosic components shown in (**c**) are the targets of degumming.

The fibre that reaches the spinning process is a technical fibre: a bundle of elementary fibres cemented by a middle lamella rich in pectin and embedded in the phloem of the stem [10] (Figure 1). Within each elementary fibre, cellulose microfibrils of high molar mass and characteristic solid-state order aggregate into the secondary wall [11]. Surrounding this cellulose core is a non-cellulosic matrix of pectin, hemicellulose and lignin, the proportion of which differs not only between species but between fractions of the same stem. In industrial hemp, for example, the bast and the woody hurd differ sharply in lignin content and in their response to chemical pretreatment [12]. Comparable variation is evident in surface free energy, which is controlled by the chemical composition of flax and hemp fibres [13], and in the structure of pectic polysaccharides extracted from three bast fibre plants [14]. Compositional heterogeneity of this kind is the reason a degumming recipe validated on one crop cannot be transplanted to another without re-optimisation.

### 1.2. The Gum System and the Requirement for Degumming

Degumming is the separation of this non-cellulosic matrix from the cellulose core. The three gum components behave differently and therefore respond to different chemistries. Pectin, concentrated in the middle lamella and primary wall, cements elementary fibres together and is consequently the primary target of any degumming route. Hemicellulose is hydrogen-bonded to the cellulose microfibrils and has to be loosened before fibre bundles can separate. Lignin, being hydrophobic and heavily cross-linked, encrusts the bundle and determines both its rigidity and its resistance to aqueous reagents.

However, the degumming process must be controlled rather than allowed to proceed to a complete removal. Chemical degumming of *Apocynum venetum* has been shown to reshape composition, structure and properties simultaneously [15], and different chemical routes applied to the same raw material yield markedly different fibre quality [16]. In ramie, degumming altered anatomical, chemical, mechanical and thermal behaviour in ways that were not monotonic with respect to gum removal [17]. Excessive severity depolymerises cellulose and lowers tenacity, whereas insufficient removal leaves residual gum that causes fibre cohesion, uneven yarns and weak interfacial bonding in composites. The operational target is thus a controlled degree of gum removal that maximises processability without sacrificing the cellulose core.

### 1.3. Scope, Sources and Positioning of This Review

This review covers the application of deep eutectic solvents (DES) to the degumming of the five bast fibre crops mentioned above. Literature was collected through structured searches of the major bibliographic databases and restricted to articles indexed in the Science Citation Index. Because the aim is a synthesis of the current state rather than an exhaustive catalogue, priority was given to studies that report quantified degumming performance, fibre properties or solvent-reuse records. Whereas recent reviews have examined DES-assisted degumming within single crops, most notably hemp [18], no comprehensive review has yet placed the five major bast fibre crops on a common comparison basis. This review addresses that gap in three ways. It compiles the DES systems, operating conditions, degumming outcomes and solvent-reuse records reported for each crop onto a uniform set of criteria. It compares neutral, alkaline and acidic DES families. It identifies where mechanistic evidence is strong and where it remains inferential.

## 2. Evolution of Degumming Technology and the Arrival of DES

### 2.1. Traditional Routes and Their Limits

Field and water retting remain the oldest and cheapest means of separating bast fibres, and they are mediated by microbial communities whose composition determines fibre quality. The processes are nonetheless slow, weather-dependent and difficult to control, as studies on hemp field retting show [19,20]. Mechanical scutching removes shives but cannot release elementary fibres from the middle lamella. Alkali boiling is the incumbent industrial route: it is fast and effective, yet it consumes large volumes of water and energy, generates strongly alkaline effluent with high organic load, and simultaneously attacks cellulose [21,22].

The burden carried by that incumbent route has been quantified for ramie, and the numbers explain the industry’s recent trajectory. Chemical degumming generates roughly 400 tonnes of effluent for every tonne of ramie processed, with the chemical oxygen demand of the degumming-stage wastewater falling between 15,000 and 25,000 mg/L [22]. The expense of treating effluent has triggered a series of economic and environmental problems, which have become the motivation for every alternative route discussed below.

### 2.2. The Spectrum of Green Degumming Routes

Among these emerging routes, enzymatic degumming has advanced furthest towards selectivity. High-specific-activity pectate lyases cloned and heterologously expressed from *Bacillus* sp. have been applied directly to ramie [23], alkaline pectinase–xylanase systems have been used to prepare hemp fibre [24], and the regulatory mechanism of pectin degradation during bacterial bio-degumming has been characterised [25]. Because enzymes are substrate-specific and operate under mild conditions, cellulose is largely preserved. The constraints are enzyme cost, batch duration and the need to combine enzymatic steps with physical or chemical pretreatments to reach industrial throughput [26], an approach pursued through ultrasound-assisted chemical–biological sequences [27] and energy-efficient enzymatic–ultrasonic routes [28].

Organic solvent and ionic liquid routes attack lignin directly, and they are effective on heavily lignified feedstocks. However, their solvent cost, recovery duty and the environmental burden of the solvent itself limit their appeal for a commodity fibre [29,30]. Where the routes have been placed side by side on a single feedstock, the spread between them is wide. Measured against chemical degumming of ramie, biological treatment requires about 50% less reagent and costs 78% less to treat its wastewater, while oxidative degumming leaves an effluent whose chemical oxygen demand is roughly 2.6 times that of the biological route. Solvent routes generate the least pollution of the three, but they remain constrained by solvent cost and inefficient recovery [22,29]. Reviews of green solvent pretreatment now treat DES as one family within this broader toolbox, not as a distinct category [31]. Across the routes surveyed, no single technology dominates on degumming efficiency, retained fibre quality, reagent cost and environmental load at the same time.

### 2.3. How DES Entered the Picture

DES were introduced as a distinct class of solvents when mixtures of choline chloride (ChCl) with urea or a carboxylic acid were shown to liquefy well below the melting points of their components [32,33]. Their adoption in biomass processing followed a different line of argument. The decisive property was not the low melting point itself, but the ability of these solvents to separate lignocellulose into its fractions: the cellulose fraction survives largely intact while lignin and hemicellulose are dissolved [34,35]. In the degumming context, DES should be positioned as a complement to, not a replacement for, alkali and enzymatic methods. That both of the recent bast fibre degumming reviews treat DES only briefly, as one option among organic-solvent methods, is itself an indication of how undeveloped this field remains [21,22].

## 3. DES Fundamentals and System Design

### 3.1. Definition, Classification and Properties

A DES is a mixture of a hydrogen bond acceptor (HBA) and a hydrogen bond donor (HBD), and its extensive hydrogen-bond network depresses the melting point of the mixture far below those of the individual components [32]. The widely used classification into types I to V is based on the nature of the HBA and HBD. Types I to IV were consolidated by Smith et al. [36], and type V, which covers non-ionic systems, was added later. The scheme is summarised in Table 1. The type III systems formed between ChCl and a hydrogen-bond-donating species dominate biomass work, and the studies on degumming rely almost exclusively on them. The physicochemical behaviour of DES, including melting point, viscosity, density, polarity, conductivity and their dependence on composition and water content, has been reviewed repeatedly [37,38,39,40].

### 3.2. Design Principles for Degumming Systems

Because the solvent must penetrate a compact, partly lignified cell wall, the first design parameter is mass transfer. The HBA/HBD pairing and the molar ratio set the hydrogen-bond density and hence the viscosity and diffusivity of the medium. The 1:2 ratio used in the original ChCl–urea (CU) system remains the most common starting point [32]. The second design parameter is acid–base character, which maps onto the three gum components. Acidic DES based on oxalic or lactic acid dissolve lignin efficiently, but the same acidity hydrolyses hemicellulose, an effect that has been identified as an overlooked cost of acidic pretreatment [41]. Alkaline systems built on ETA-type donors favour the removal of pectin and hemicellulose and are comparatively gentle on cellulose, whereas neutral systems based on urea, glycerol or ethylene glycol are the mildest and, in practice, the most readily recycled. Hydrophobic DES constitute a separate branch whose value in aqueous fibre processing is limited [42]. Because the desirability of a solvent is bound up with the fractionation of polysaccharides, the selection of HBA–HBD pairs is best treated as a targeted separation problem [43,44].

### 3.3. Questioning the “Greenness” of DES

DES are routinely described as green solvents on the grounds of their low volatility and low toxicity, and these properties are genuine. However, in the strict sense, greenness is a property of the whole life cycle: it turns on how the solvent is manufactured, on the burdens that synthesis carries, and on its environmental fate and disposal. It is nevertheless an incomplete basis for choosing a degumming medium. Viscosity in the neat state is high enough that water or a co-solvent is often added in practice, which changes the solvent’s own hydrogen-bond network and with it the degumming outcome, a variable that is recorded as a process parameter and never controlled as a chemical one. The physicochemical reviews of DES record that water content shifts melting point, viscosity, conductivity and polarity [37,38,39,40]. In pretreatment, a DES-to-water ratio of 1:5 was calculated to build a new hydrogen-bond network more favourable to the interaction with lignocellulose [45]. Within degumming itself, the record is much thinner. The flax pretreatment was run in 90% DES [46]. Water content is thus one of the variables that decides how far the solvent penetrates a compact cell wall, yet no other study among the eighteen compiled here reports the water content of its bath. Recovery, moreover, is usually achieved by precipitation with an anti-solvent. A process that recycles its DES therefore still consumes water: the solvent is recovered, but the water demand and the regeneration duty remain.

The decisive test is therefore not the solvent’s material properties but the balance of the whole process, and for degumming that balance has rarely been calculated. Published degumming studies report gum removal, fibre properties and, in a minority of cases, reuse over a few cycles, but almost none report the energy and water consumed in regenerating the solvent or the fate of the dissolved lignin and hemicellulose as products. Until such balances exist, the claim that DES degumming is greener than alkali boiling rests on the mildness of the operating conditions alone.

## 4. DES Degumming of the Five Bast Fibre Crops

**Figure 2:**
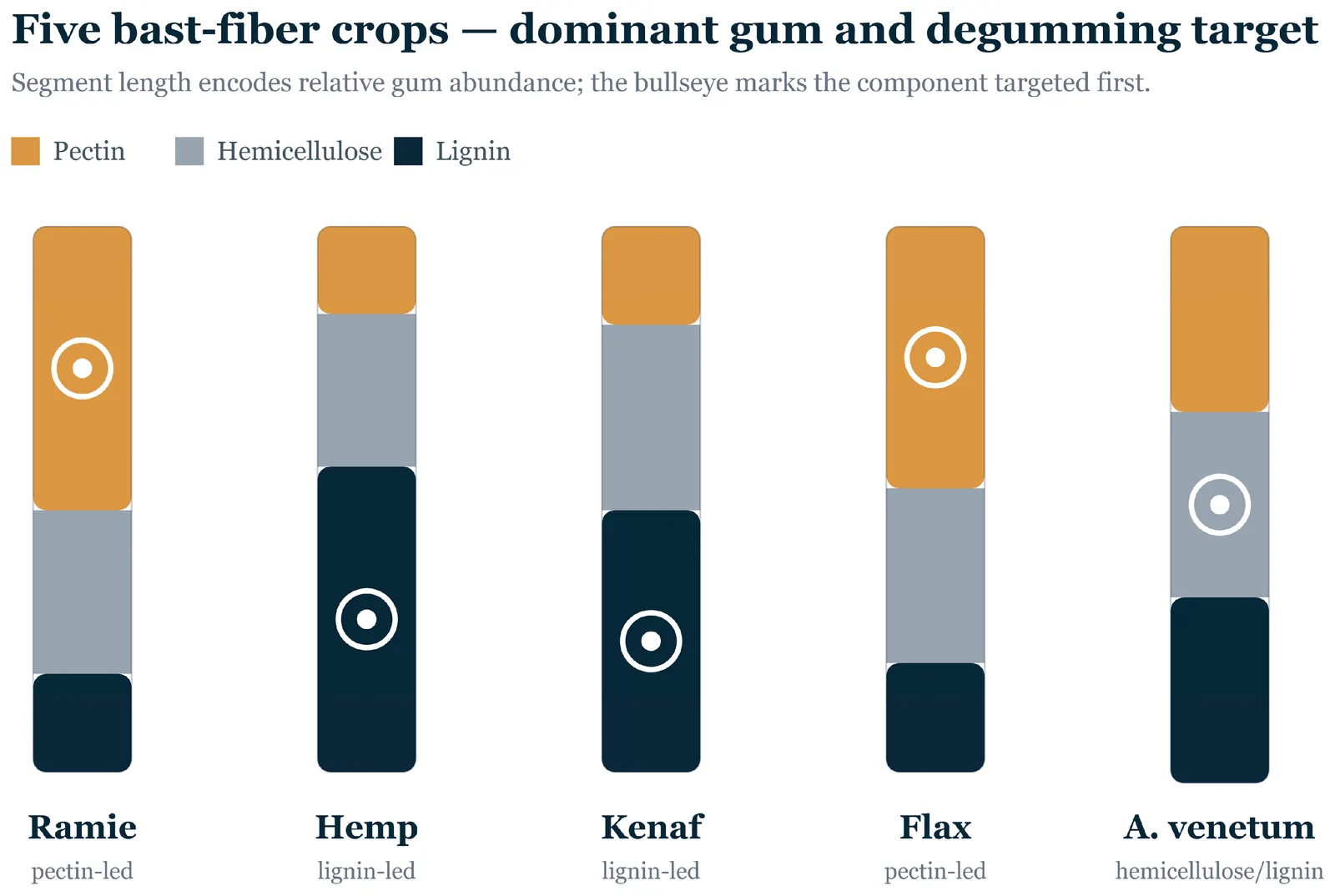
Dominant gum fraction and primary degumming target across the five bast fibre crops. Each vertical bundle is divided into three stacked segments, whose lengths are proportional to the relative abundance of pectin, hemicellulose and lignin. The concentric target marks the fraction that each crop is primarily degummed to remove: pectin for ramie and flax, lignin for hemp and kenaf, and hemicellulose and lignin for *Apocynum venetum*.

The five crops surveyed in this section differ in the composition of the gum that must be removed and in the component that each degumming route primarily targets (Figure 2).

### 4.1. Ramie

Ramie is the crop on which DES degumming is most mature. Six studies published between 2019 and 2026 follow a continuous line of inquiry, from a single-step boiling process through the deliberate protection of cellulose during treatment to solvent recycling, and no other bast fibre crop has been followed this far along the chain. This early start has two explanations, one technical and one commercial. The technical reason is the composition of the fibre. Ramie bast contains 65–75% cellulose and only 0.8–1.5% lignin [22], so the gum that has to be removed, which accounts for roughly a quarter to a third of the fibre mass, consists mainly of hemicellulose and pectin. The intercellular layer of ramie is likewise rich in pectin and in mannan and xylan. Ramie is therefore easier to degum than lignin-rich fibres and does not require harsh conditions [22]. A gum of this kind is the most favourable substrate for a solvent whose principal action is hydrogen-bond disruption. The commercial reason is the state of the industry. Ramie is a long-established textile fibre with a large processing sector, and the pollution load of that sector is a recognised problem. An alkali-free or low-alkali route for this crop therefore answers an immediate industrial need.

#### 4.1.1. The Alkali-Free, Single-Step Paradigm

The founding result is a one-step boiling process. Huang et al. [47] screened three DES, namely CU, choline chloride–imidazole (CI) and ethylamine hydrochloride–ethylene glycol, and showed that all three removed most non-cellulosic material from raw ramie in 2 h in a single step without any alkali and without damaging the cellulose structure. The three systems were not equivalent. CU-treated fibres gave the highest tenacity (6.51 cN/dtex) at a residual gum content of 3.76%, a level the authors described as close to that of traditionally alkali-treated fibres. CI-treated fibres were the most extensible, reaching a break elongation of 3.30% at a tenacity of 5.50 cN/dtex. Ethylamine hydrochloride–ethylene glycol removed the most gum of all (3.15% residual) yet produced the weakest fibres (3.05 cN/dtex). This last comparison is the most instructive result in the study: it demonstrates that gum removal and mechanical quality do not co-vary; a low residual gum content alone cannot be regarded as proof of satisfactory degumming performance. The same study also reports that ethylamine hydrochloride–ethylene glycol and CI remove lignin more effectively than CU according to Raman mapping results, indicating that the three solvent systems differ in their preferential targets among fibre components.

A second route achieved comparable fibre quality without any alkali, but at the cost of an added physical pretreatment. Song et al. [48] combined steam explosion with a DES treatment and reported a residual gum content of 4.68%, an average fineness of 1420 Nm and a breaking tenacity of 5.98 cN/dtex, all of which met the requirements of the relevant Chinese national standards and were comparable to the traditional alkali process. Fourier transform infrared spectroscopy, scanning electron microscopy and X-ray diffraction jointly confirmed the degumming effect. Two aspects of this result deserve note. First, the reported performance is obtained using a fully alkali-free process. Second, the DES step runs under mild conditions: CU mixture at a 1:2 molar ratio for degumming at 100 °C for 2 h, meaning the degumming load is effectively split between the solvent and steam explosion. “Alkali-free” therefore does not mean “pretreatment-free”, a distinction that matters when the process is transferred to crops whose gum is less tractable.

#### 4.1.2. Quality Control: Cellulose Protection and the Turn to Alkaline Systems

Once the one-step process was established, the main problem shifted from gum removal to cellulose retention. Ramie cellulose is readily degraded when the reaction is intense and the conditions are not properly controlled, which shows up directly as a loss of mechanical performance [49]. The response was to add a sacrificial protectant. Anthraquinone at only 0.2 g/L raised the degree of polymerisation (DP) by 3.2% and the crystallinity by 5.56%, and increased breaking tenacity, breaking elongation and work of rupture by 8.44%, 11.9% and 33.3% respectively. The additive also raised the degradation temperature, accelerated lignin removal and protected carbohydrates from excessive degradation. The significance of this result is methodological, not merely incremental: it separates two objectives that the earlier single-variable screening had conflated, and it shows that the severity needed to remove gum and the severity that cellulose can tolerate are not the same point.

A parallel development moved the solvent itself towards alkalinity. Song et al. [50] built an alkaline DES from ChCl and monoethanolamine at a molar ratio of 1:8 and established 140 °C as the optimum degumming temperature, selecting it through a composite evaluation of fibre yield, residual gum content, fineness and breaking tenacity. The resulting single fibres contained minimal residual hemicellulose and lignin, showed improved crystallinity and thermal stability, and were comparable to conventionally alkali-treated fibre, with the additional advantages of reduced processing time and reduced chemical consumption. A more recent study from the same group replaced the amine donor with ethylene glycol and promoted the system with a small amount of alkali: ChCl and ethylene glycol at 1:6 with 0.6% NaOH at 140 °C, optimised against residual gum content as the sole target indicator [51]. Lignin and hemicellulose were significantly removed, and the fibres reached a higher crystallinity index and maximum degradation-rate temperature than those obtained by the earlier route, again meeting the national standard for refined ramie.

#### 4.1.3. Solvent Recycling

Because the DES reagents are costly and their recovery has been inefficient, reuse has been treated in the ramie literature as a precondition for application, not as an optional refinement [52]. Three studies address it, and they adopt two different strategies. The first is solvent regeneration: Huang et al. [47] reported that their DES could be reused repeatedly, and that fibres obtained after the fifth cycle of treatment still met the requirements of downstream applications. Lin et al. [52] examined reuse systematically, tracking surface morphology, chemical composition, crystallinity, thermal stability, DP and tenacity as the number of reuse cycles increased. Over the range studied, crystallinity lay between 65.7% and 70.1%, the thermal decomposition temperature between 353 °C and 356 °C, and the DP between 1679 and 2445, while the reusable solution continued to remove the majority of the non-cellulosic components.

The second strategy is component replenishment instead of solvent regeneration: the spent liquor from the alkali-promoted system was reused after adding fresh NaOH and was shown to retain its degumming capacity [51]. Replenishment is often considered the cheaper option because it avoids a full regeneration step, but it converts the solvent from a recyclable medium into a consumable that requires alkali make-up each cycle. Which of the two strategies is superior depends on the energy and water demands for solvent regeneration, yet none of the three included studies quantifies this trade-off.

### 4.2. Hemp

#### 4.2.1. Why Hemp Sets Different Operating Conditions from Ramie

Hemp carries the largest body of DES degumming work of any single crop examined in this review, with eight studies that include the only dedicated review of DES degumming for one bast fibre crop [18]. The reason its operating window differs from that of ramie is compositional. Hemp bast contains more lignin than ramie. This makes the fibre more resistant to the hydrogen-bond disruption on which neutral DES depend, and leads the dedicated review to identify semi-degumming, the deliberate retention of part of the gum in order to preserve fibre integrity, as the appropriate target in place of exhaustive removal [18]. The conditions reported for hemp are correspondingly more severe than those reported for ramie: the highest temperature in the present survey of all five crops, 175 °C, appears only in hemp work [53], whereas the ramie routes described above operate between 100 and 140 °C.

#### 4.2.2. The DES–Alkali H_2_O_2_ Combination and Its Mechanistic Evidence

The most mechanistically detailed hemp study reports a process that is not DES alone. Ma et al. [54] combined a CU DES (1:2) with an alkaline hydrogen peroxide step in an integrated route that the authors designate DAHD, and characterised the product by pyrolysis–gas chromatography/mass spectrometry (Py-GC/MS) and two-dimensional nuclear magnetic resonance (2D-NMR). Removal of non-cellulosic components reached 53%. Tenacity, fibre length and DP were 25%, 30% and 20% higher, respectively, than for fibre degummed by the conventional alkali route, and the cellulose content of the treated fibre exceeded that of the alkali-treated control. The mechanistic evidence is correspondingly specific: the principal cellulose pyrolysis product (levoglucosan) increased by 27.8%, ester and ketone contents decreased by 1.07% and 11.1% as hemicellulose was removed, and 2D-NMR revealed significant cleavage of β-O-4′ linkages with β-5′ linkages eliminated entirely. G-type lignin was degraded more extensively than S-type. The DES also retained adequate degumming performance after reuse cycles. Two caveats to the reported findings merit follow-up research. First, the developed process is a DES-integrated hybrid system, not a standalone DES treatment. Second, although the resulting fibre outperformed the alkali reference in tenacity, staple length and DP, the reported 53% removal rate of non-cellulosic components is only moderate in absolute terms.

#### 4.2.3. Microwave Assistance and Ternary Systems

Microwave energy has been used to shorten treatment time. Ahmed et al. [55] combined microwave energy with DES and compared the product against alkali-treated fibre. Holocellulose content reached 98.6% at a 1:20 fibre-to-DES ratio, comparable with the 98.9% of the alkali-treated control. The reduced enthalpy of the treated fibre (171 J/g, against 272 J/g for raw hemp) was attributed to the removal of the gummy material. Ultraviolet protection factors of 118 and 114 were obtained at fibre-to-DES ratios of 1:10 and 1:20. The same group extended the work in a companion study in which several DES were screened in combination with microwave energy and without any further treatment: residual lignin fell to 2.88%, crystallinity reached 83.3% for the CU system, and the maximum degradation temperature of the treated fibre was 356 °C [56].

Ternary systems have been used to lower the temperature requirement. Haque et al. [57] compared two ternary DES, ChCl/lactic acid/glycerol and ChCl/lactic acid/urea both at 1:5:2, against the binary ChCl/lactic acid system and a traditional degumming route. When applied at 105 °C for 2 h with a bath ratio of 1:20, the ternary systems efficiently removed non-cellulosic components. This treatment yielded better fibre separation, increased crystallinity and higher tenacity relative to the conventional process, while requiring a shorter processing duration. Structural and thermal analyses confirmed that cellulose integrity was preserved. Solvent recovery and reuse were demonstrated for at least four cycles, and a calculation of chemical, energy and water costs over those four cycles showed the DES process to be more economical than the traditional route.

#### 4.2.4. Systematic Screening and Process Modelling

Two recent studies move hemp work from single-system demonstrations towards screening and prediction. Gao et al. [53] prepared three systems, CU, ethylenediamine hydrochloride–ethylene glycol and CI, and applied them to seed hemp. All three removed pectin, hemicellulose and lignin in the amorphous region without damaging the structure or crystalline morphology of the cellulose, increasing the cellulose fraction from 54% to about 80%. The systems differ in selectivity: CI at a molar ratio of 3:8, 175 °C for 2.5 h, removed hemicellulose and pectin most effectively. Ethylenediamine hydrochloride–ethylene glycol at 1:2, 175 °C for 3 h, removed lignin most effectively. CU, the lowest-cost system, gave a balanced removal of all three non-cellulosic components, which the authors identify as probably the optimal choice for industrial production. Density functional theory (DFT) was used to examine the configurational stability of the three systems.

Firouzi et al. [58] approached the same problem statistically. Three DES were screened for cellulose extraction from hemp bast, ChCl–glycerol was selected for its superior performance, and four process parameters (treatment time, temperature, DES-to-hemp ratio and glycerol-to-ChCl ratio) were optimised using a central composite design within a response surface methodology. Five machine-learning algorithms were then applied to the response surface methodology output to predict the extraction process, with the random tree model performing best at a predicted R^2^ of 0.855. SEM, FTIR, thermogravimetric analysis and XRD confirmed the removal of impurities. The crystallinity index was 81.4%, and the cellulose yield rose from 58.1% in untreated hemp to 69.7% in the optimally treated sample.

The single dedicated review focused on hemp [18] concludes the literature analysis for this crop. It states that CU systems achieve cellulose retention above 90% alongside nearly complete lignin removal at moderate temperatures, and hybrid strategies reduce processing time by 50%. That review is limited exclusively to hemp; accordingly, its comparisons are confined to different treatments for hemp and do not extend across crops (Section 1.3).

### 4.3. Kenaf

Kenaf bast has a high lignin content, which accounts for the particular difficulty of conventional chemical degumming of this feedstock [59]. Only three studies in the period surveyed apply DES to kenaf, and they describe three different process concepts. The first is a screening of hydrogen bond donors that established the alkaline amide system. The second is an alkaline amine system designed for repeated recycling without purification. The third is a combined microwave-alkaline/ultrasonic route aimed at cutting reagent, water and time consumption. All three come from the team that also produced the ramie and *Apocynum venetum* work discussed in Section 4.1 and Section 4.4.

#### 4.3.1. Screening of Hydrogen Bond Donors and the Proposed Mechanism

Nie et al. [60] synthesised DES from one hydrogen bond acceptor and three representative hydrogen bond donors and compared them on kenaf bast, assessing morphology, chemical composition, crystallinity, DP and physical properties. The alkaline amide system CU gave the best degumming effect, and the optimum conditions were 160 °C for 2 h. The mechanism proposed by the authors has three steps, in which chloride competes for the hydrogen bonds of the assembly, the solvent deprotonates phenolic hydroxyls to dissolve lignin, and β-O-4 bonds are then cleaved.

#### 4.3.2. An Alkaline DES That Survives Repeated Recycling

Song et al. [59] took a different route, using the alkaline amine system choline chloride–ethanolamine (ChCl–ETA) on kenaf bast. Conditions were optimised on the basis of residual gum content, and the reported optimum was a ChCl:ETA ratio of 1:6 at 120 °C. The distinguishing result lies in recycling: recycled ChCl–ETA mixtures retained a degumming capacity comparable to that of the conventional double-boiling method for at least five degumming cycles without any purification, as measured by the residual gum content of the treated fibres. Chemical composition analysis was supported by SEM, FTIR, XRD and thermogravimetric analysis, with two-dimensional NMR spectroscopy included among the characterisation methods. The authors cite the process’s low pollution and high efficiency as the basis for industrial-scale application.

#### 4.3.3. Process Intensification and the Resource Savings Claimed

The earliest of the three kenaf studies [61] combined DES treatment with microwave and alkaline-ultrasonic stages. The refined dry fibres obtained had a residual gum content of 9.42%, a fineness of 4.13 tex and a breaking tenacity of 13.7 cN/tex, with a crystallinity index of 66.7%. These values were reported as comparable to those of fibres produced by the traditional two-step alkali boiling method. The authors expressed the process advantage in resource terms: compared with that reference route, the combined treatment reduced chemical consumption by 48.9%, water consumption by 66.7% and processing time by 66.8%.

### 4.4. Flax and Apocynum venetum

#### 4.4.1. Flax: DES as a Pretreatment for Microbial Degumming

Flax is represented by a single study in this review. Xiang et al. [46] treated flax roving with a CU DES pretreatment followed by microbial treatment, with orthogonal tests giving an optimum pretreatment of 100 °C for 120 min in 90% DES. Relative to fibres treated with microorganisms alone, the combined process gave better breaking tenacity, 16.8 cN/tex, and average removal rates of approximately 86.4% for pectin, 26.7% for hemicellulose and 55.1% for lignin. Pectin was thus removed far more completely than the other two components. NMR analysis indicated that the composition of the DES was not damaged during pretreatment, which the authors take to imply that the bulk solution could be reused, although no recycling experiment is reported.

#### 4.4.2. *Apocynum venetum*: The Earliest DES Degumming of a Bast Fibre

Song et al. [62] applied DES to the bast of *A. venetum*, and this is the earliest DES degumming study in the review. The process was two-stage: a microwave-assisted pretreatment with a 1:2 CU mixture, optimised to 60 min at 110 °C, followed by alkaline degumming. Chemical composition, surface microstructure, cellulose crystallinity, DP and thermal properties were measured on both the semi-degummed and the refined dry fibres. The combined treatment removed hemicellulose and lignin and gave a smooth, clean fibre surface. The authors also report that it extracted amorphous cellulose, which raised fibre crystallinity and lowered the average cellulose molecular weight. Thermal stability improved markedly. The refined dry fibres were stronger than conventionally twice-boiled alkaline fibres, at 14.1 cN/dtex against 11.2 cN/dtex. The authors note that recycling and reuse of the DES remained to be evaluated in future work.

## 5. Evaluation and Characterisation of DES Degumming

### 5.1. Indicators of Degumming Performance

#### 5.1.1. Residual Gum, Removal Rate and Yield

Residual gum content is the primary quality indicator within China’s bast fibre industry and the most frequently reported parameter in this review. The compiled residual gum values range from 3.15% to 9.42%. Specifically, the ethylamine hydrochloride–ethylene glycol system achieved 3.15% for ramie, while the CU system in the same screening test attained 3.76% [47]. An alkali-free steam-explosion process for ramie yielded a residual gum content of 4.68% [48], and a combined DES–microwave–alkaline ultrasonic treatment for kenaf produced a value of 9.42% [61].

However, a large portion of the literature quantifies degumming performance using alternative metrics. Ma et al. [54] reported a single overall removal rate of 53% for total non-cellulosic components. Xiang et al. [46] provided component-wise removal data; their values vary markedly within the same experiment, reaching 86.4% for pectin, 55.1% for lignin, and merely 26.7% for hemicellulose. Gao et al. [53] reported neither removal nor residual but the composition of the product, cellulose rising from 54% to about 80%. Ahmed et al. [55] measured holocellulose content and obtained a value of 98.6%, compared with 98.9% for the alkali control. Since holocellulose includes hemicellulose, this result cannot be directly compared with the cellulose contents mentioned above. Firouzi et al. [58] reported a cellulose-to-lignin mass ratio of approximately 70.8 to 17. Yield introduces another discrepancy in measurement basis: Lin et al. [52] presented fibre yields ranging from 74.2% to 78.6%, while Firouzi et al. [58] provided cellulose yields that increased from 58.1% to 69.7%. The same outcome is therefore recorded in several forms, and they cannot be interconverted without the composition of the starting material, which is not always reported.

#### 5.1.2. Fineness and Breaking Tenacity

Fineness and breaking tenacity are quoted in the unit system customary in each study. For ramie, fineness is given as metric count, 1420 Nm [48], and as decitex, 6.30–7.87 dtex [52], while for kenaf it is given as tex, 4.13 tex [61]. Tenacity is quoted in cN/dtex for ramie and A. venetum, namely 6.51, 5.50 and 3.05 cN/dtex for the three systems screened by Huang et al. [47], 5.98 cN/dtex [48], 5.21–6.72 cN/dtex [52] and 14.1 cN/dtex for refined A. venetum fibre against 11.2 cN/dtex for its alkali control [62]. For kenaf and flax it is quoted in cN/tex, 13.7 cN/tex [61] and 16.8 cN/tex [46]. These units interconvert exactly through their definitions, so their plurality reflects national convention and does not obstruct comparison. The measured analyte is not always clearly stated; this represents an oversight in reporting instead of a discrepancy in underlying principles. Two standards in routine use make the distinction explicit: ISO 1973:2021 [63] specifies a gravimetric method applicable to bundles of fibres and a vibroscope method applicable to individual fibres, and notes that its results are less precise for natural than for man-made fibres. ASTM D3822/D3822M-14 [64] covers single fibres only and states that tensile strength increases as specimen length decreases, so that the gauge length must be reported if values are to be comparable. No study in this review says whether its fineness refers to a bundle or to an elementary fibre, and none states the level at which the tensile test was performed or the gauge length used. This distinction matters because the level at which a property is measured changes its value: for flax, single fibre, yarn and unidirectional fabric prepared from one feedstock give different properties [65]. Only if the test object and the gauge length are named can the printed values be used directly by others. Length is reported most thinly of all, as a relative gain of about 30% over alkali treatment [54] or as equivalence to the alkali control [62].

### 5.2. Compositional and Structural Characterisation

#### 5.2.1. Chemical Composition

Selectivity is argued from chemical composition: how much cellulose, hemicellulose, lignin and pectin the fibre contains before and after treatment. The four fractions are not obtained in the same way. Lignin is usually reported as acid-insoluble (Klason) lignin [66], the structural carbohydrates by two-step acid hydrolysis [67], and hemicellulose often by difference, as holocellulose minus cellulose. These routes do not return the same value for the same sample, so a figure for hemicellulose depends on the route that produced it.

Composition is also not the only evidence used for selectivity. Huang et al. [47] ranked the selectivity of their three systems from Raman mapping, not from composition, a different kind of evidence with a different spatial basis. Nie et al. [60] and Xiang et al. [46] reported component-wise removal against an untreated reference, and the composition of that reference is not always reported for the same variety. The hemp routes aim at controlled retention as opposed to complete removal, that is, semi-degumming to keep the fibre intact [18].

#### 5.2.2. Instrumental Characterisation

FTIR is almost universally employed and interpreted qualitatively: spectra are used to show that the absorption bands associated with lignin and pectin weaken or disappear, a useful confirmation but not a measurement. The largest quantitative divergence appears in XRD. Crystallinity is obtained by the Segal method, by deconvolution of the diffraction pattern, or indirectly from FTIR band ratios, and the three procedures do not return the same value for the same sample. Nam et al. [68] assessed the applicability of the Segal approach, Zhang et al. [69] obtained crystallinity from micro-FTIR with curve fitting, and Montoya-Escobar et al. [70] estimated it from XRD and FTIR jointly by Fourier series. Reported values here range from 65.7 to 70.1% [52] and 66.7% [61] up to 81.4% [58] and 83.3% [56], while other studies report only the direction of change, an increase of 5.56% for the anthraquinone route [49] or a bare statement that crystallinity improved [50,51]. A spread from about 66% to about 83% across studies therefore reflects the method of determination as well as the chemistry.

Thermal analysis shows the same pattern in a different index. Lin et al. [52] reported a decomposition temperature of 353–356 °C, Ahmed et al. [56] a maximum degradation temperature of 356 °C, and Ahmed et al. [55] a change in enthalpy from 272 to 171 J/g. Other studies state only that thermal stability improved. The indices in normal use for cellulosic natural-fibre systems are set out in the thermal-characterisation literature [71,72], but none has been adopted here, and an enthalpy of transition is not comparable with a decomposition temperature. DP is reported as an absolute range of 1679–2445 in one study [52] and as a relative gain of 20% in another [54]. Zhou et al. [73] showed that a high DP depends on retaining the native structure, which makes this index the most sensitive of the set to both process and measurement. SEM images of surface morphology are widely reported, yet rarely quantified. Hao et al. [74] set out the analytical-pyrolysis practice on which such quantification rests. This is a methodological reference rather than a source of degumming evidence. Almost every study describes smoother, cleaner fibre surfaces, but these findings are only meaningful when benchmarked against untreated or alkali-treated reference samples.

#### 5.2.3. Bond-Level Characterisation

Only two of the eighteen research studies collected here provide bond-level evidence. Ma et al. [54] applied Py-GC/MS and two-dimensional NMR to hemp treated by the combined DES–alkaline H_2_O_2_ route: levoglucosan yield from cellulose rose by 27.8% while esters fell by 1.07% and ketones by 11.1%. β-O-4′ linkages were substantially cleaved, β-5′ linkages disappeared, and G-type lignin units were degraded more extensively than S-type. Song et al. [59] included two-dimensional NMR in the characterisation of the recycled kenaf system. These two studies are the only ones that make a claim about which bonds break instead of about how much material was removed, and between them they cover two of the five crops. The mechanistic discussion in Section 6 thus rests on linkage-level evidence from two laboratories, and the consequent inter-crop data imbalance is addressed in Section 6.1 and Section 8.2.

## 6. Mechanism Consensus and Cross-Crop Comparison

### 6.1. Mechanism Consensus and Divergence

#### 6.1.1. The Consensus Pathway

The mechanistic account that has become standard in this literature is a three-step sequence. Chloride ions compete for the hydrogen bonds that hold the fibre assembly together and weaken them. The alkaline DES then removes lignin by deprotonating phenolic hydroxyl groups. Finally, β-O-4 linkages are cleaved, releasing lignin fragments. The sequence was proposed for kenaf bast [60], and each step has independent support from pretreatment work on wood and agricultural residues. For the first step, molecular dynamics shows that the chloride anion interacts with lignin with an average energy comparable to that of the hydrogen bond donor, so the anion is a participant in dissolution, not a counter-ion [75]. A simulation of DES–lignocellulose hybrids found that CU disrupted 78.3% of the hydrogen bonds between cellulose and a glucuronoxylan and 68.4% of those between cellulose and a lignin model dimer, more than the glycerol- or lactic-acid-based systems tested alongside it [45]. For a benzyltriethylammonium chloride system, the chloride–lignin interaction is predominantly electrostatic, while the cation acts through van der Waals contacts and displaces the native π–π stacking of lignin [76]. For the third step, the proton-donating hydroxyl and carboxyl groups of acidic donors cleave ether bonds and part of the carbon–carbon bonds with them [77], and acidic DES are reported to disrupt β-O-4 and ester linkages across a wide range of feedstocks [78]. The second step is the weakest of the three. Deprotonation of a phenolic hydroxyl presupposes an alkaline medium, so it describes the ETA and monoethanolamine systems and the alkali-promoted ones, but not the neutral CU family that supplies most of the degumming literature. The sequence as published is best read as a mechanism of alkaline DES that has been extended, without testing, to the neutral family.

#### 6.1.2. Selectivity Among Lignin Units

The established reaction pathway identifies the types of chemical bonds that cleave. A separate unresolved question concerns which lignin subunits are preferentially attacked, and three literature sources address this issue. Two-dimensional NMR of hemp treated by the DES–alkaline H_2_O_2_ route showed G-type units degraded more extensively than S-type [54], and in the ethanol-assisted ChCl–lactic acid system the condensed G unit fell from 7.23% to 0.87% while J and S′ substructures were removed altogether [79]. A machine-learning analysis of ternary DES pretreatment approached the same question from a different direction. There, the syringyl-to-guaiacyl ratio appeared among the structural parameters that predict depolymerisation, and temperature, the polarity of the hydrogen bond donor and the acidity of the Lewis acid were identified as the dominant process variables [80]. The convergence is suggestive but not independent, since it rests on one NMR observation in hemp, one in a wood lignin and one statistical model, and its practical consequence has not been measured in any of the five crops. If G units are consumed preferentially, the lignin remaining in the spent liquor would be expected to be enriched in S units and to differ in reactivity from lignin recovered from a fully delignified feedstock.

#### 6.1.3. Uneven Depth of Mechanistic Evidence Across Crops

**Figure 3:**
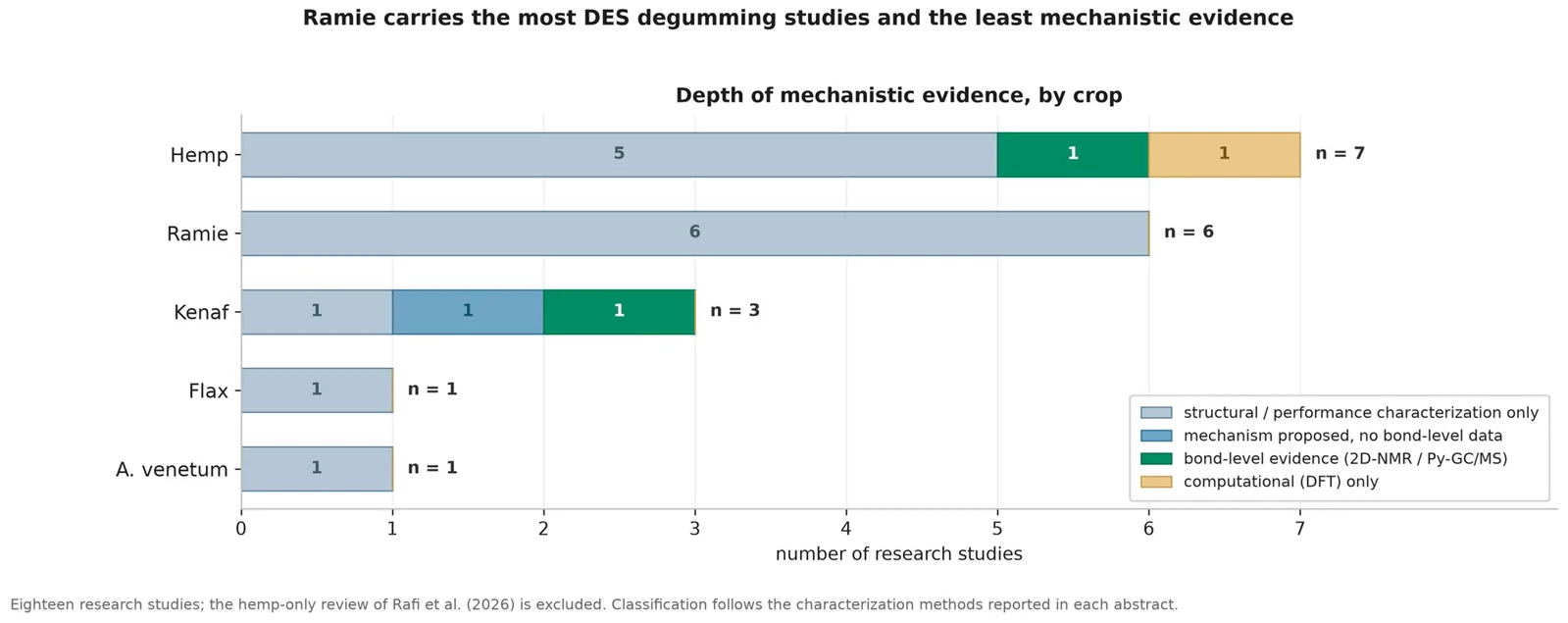
Depth of mechanistic evidence across the five crops, by study. Eighteen research studies are classified by the deepest level of mechanistic evidence reported in their abstract: structural or performance characterisation only, a mechanism proposed without bond-level measurement, bond-level evidence from 2D-NMR or Py-GC/MS, and computational DFT comparison without connection to the fibre. The hemp-only review of Rafi et al. [18] is excluded. Ramie, the crop with the largest number of DES degumming studies, has none at bond level.

Table S1 and Figure 3 make the imbalance explicit. Of the eighteen research studies, two provide bond-level evidence: the hemp work that coupled Py-GC/MS with two-dimensional NMR and traced β-O-4′ cleavage and the elimination of β-5′ linkages [54], and the kenaf study that included two-dimensional NMR in the characterisation of its recycled solvent [59]. One proposes the three-step mechanism without bond-level measurement of its own [60], and one uses DFT to compare the configurational stability of three systems without connecting the result to the fibre [53]. For ramie, flax and *Apocynum venetum*, the evidence stops at FTIR, XRD, SEM and thermal analysis, none of which identifies a bond. Consequently, the bast fibre crop with the most extensive research on DES degumming lacks any bond-level mechanistic evidence. A mechanism widely regarded as established within this field relies on data from only two laboratories, covers merely two out of the five bast fibre crops, and has not been validated for the crop that accounts for one third of relevant studies. This represents the mechanistic parallel to the evaluation issue addressed in Section 8.2. In both scenarios, conclusions are drawn at a finer structural scale than the available measurements can substantiate, and there is no universally accepted criterion defining the required resolution of supporting evidence in either case.

### 6.2. Comparison of DES Families

**Figure 4:**
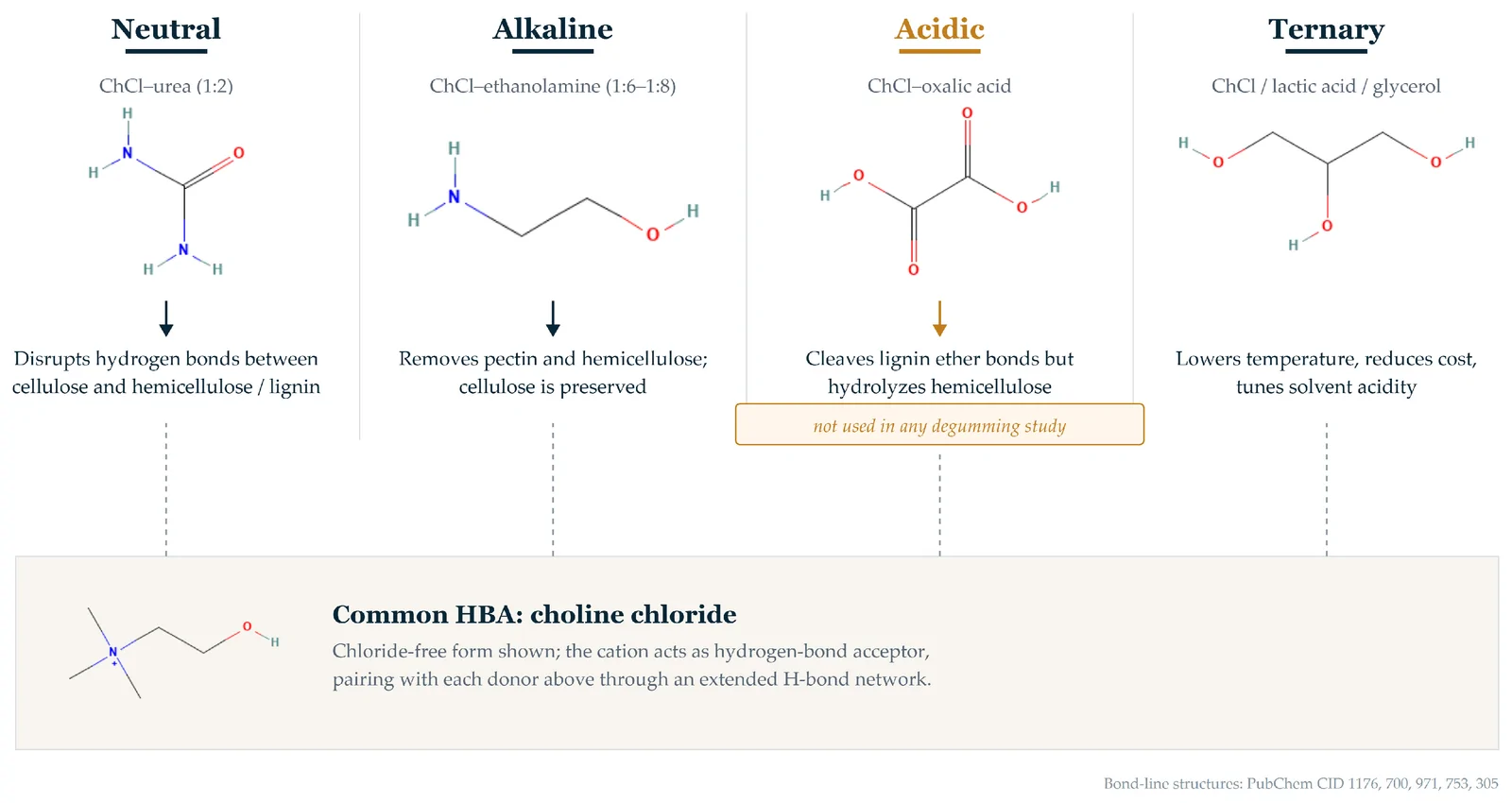
Principal DES families and their roles in gum removal. Bond-line structures of the representative hydrogen bond donor of each family are shown; the common hydrogen bond acceptor, ChCl, is drawn at the bottom. Neutral systems disrupt the hydrogen bonds between cellulose and hemicellulose or lignin; alkaline systems remove pectin and hemicellulose while preserving cellulose; acidic systems cleave lignin ether bonds but also hydrolyse hemicellulose, and are therefore not used in any degumming study. Ternary systems lower the operating temperature and cost and tune the solvent acidity.

The DES systems compiled here fall into four families whose donors act on the gum by distinct chemical routes and therefore remove different components (Figure 4).

#### 6.2.1. Neutral Systems

Neutral systems built on urea, glycerol or ethylene glycol dominate the literature and are the mildest of the three families. CU gave the highest tenacity at a residual gum content of 3.76% in the ramie screening that opened the field [47], and it was again the best of the donors tested on kenaf [60]. On hemp it was neither the strongest on hemicellulose and pectin, nor on lignin, but the most balanced across the three non-cellulosic components and the cheapest, which its authors read as the reason it is probably the industrial choice [53]. The family also has the strongest computational support, with the urea system disrupting hydrogen bonds across cellulose–hemicellulose and cellulose–lignin hybrids more effectively than the glycerol- and lactic-acid systems tested beside it [45], and with a donor’s functional groups and oxygen count identified as the determinants of its hydrogen-bond network with lignin [75]. Diol-based systems of this family have separately been shown to fractionate biomass while stabilising the lignin released [81]. One qualification warrants a caveat: a neutral donor does not guarantee a neutral process. The most recent ramie route uses ethylene glycol with 0.6% NaOH and is alkaline in operation whatever its donor is called [51].

#### 6.2.2. Alkaline Systems

Alkaline systems are defined by the donor instead of by any added base. ChCl–monoethanolamine at 1:8 [50] and ChCl–ETA at 1:6 [59] are the two that have been optimised as amine systems in their own right. Their appeal is threefold: they act on the pectin- and hemicellulose-dominated gums of ramie and kenaf while leaving cellulose comparatively intact; they hold the longest reported recycling records, five cycles without purification on kenaf; and they operate at 120–140 °C, below the most severe hemp routes. The first generation of ramie DES degumming was presented as an alkali-free proposition, and the field has since moved back towards alkali, whether through an amine donor or through deliberate NaOH addition. The 0.6% NaOH of the newest system is low by the standards of alkali boiling. Its authors still describe the route as less polluting and as enabling low alkali utilisation, so the environmental claim is not abandoned. The claim now concerns how much alkali is used, not whether it is used at all.

#### 6.2.3. Acidic Systems

Binary acidic DES built on oxalic or lactic acid are the most powerful delignifying solvents in the wider pretreatment literature, with lignin removal or extraction between 96% and 99.6% reported for several feedstocks [78], yet not one of the eighteen degumming studies uses one. The reason lies in the target, not the solvent itself. Lignin is a minor component of the gum in ramie and flax, so a solvent optimised for delignification addresses the smaller part of the problem. The acidity that cleaves lignin ether bonds also hydrolyses hemicellulose, an effect identified in the pretreatment literature as an overlooked cost [41] and one that conflicts with the requirement to keep the cellulose core intact. Lactic acid does appear in degumming, but only as one donor of a ternary system in which an amide or alcohol co-donor tempers the acidity [57].

#### 6.2.4. Ternary and Mixed-Solvent Systems

Ternary and mixed solvent systems serve two distinct functions in fibre degumming. The first is to reduce temperature or solvent cost: the ChCl/lactic acid/glycerol and ChCl/lactic acid/urea systems removed non-cellulosic material at 105 °C, well below the 140 °C of the alkaline ramie optima, and were shown over four cycles to cost less than the traditional route [57]. The second is to tune the solvent itself. Water and co-solvents are treated as variables as discussed in Section 3.3. Machine learning has begun to be applied to this design space, with temperature, donor polarity and Lewis-acid acidity emerging as the controlling variables in a model that predicted depolymerisation outcomes with a test-set coefficient of determination above 0.76 [80].

### 6.3. Common Patterns and Their Limits

#### 6.3.1. The Apparent Universality of CU

CU is the most frequently used system and the one most often identified as optimal, and the pattern deserves precise statement, not repetition as a general finding. It was selected on ramie against two other systems [47] and on kenaf against three donors [60]. As noted for hemp above, it also has the strongest independent computational support, from a group not involved in any of the degumming studies, where it disrupted more hydrogen bonds across lignocellulosic hybrids than the glycerol- and lactic-acid systems tested with it [45]. Three qualifications limit the claim. In the ramie screening it was not best on every index, since the ethylamine hydrochloride–ethylene glycol system removed more gum. Seven of the eighteen research studies come from one team, so its recurrence in the record reflects a shared experimental lineage as well as independent selection. No study has yet compared a neutral system with an alkaline one at matched conditions on the same crop, so it remains open whether urea systems are genuinely better or merely earlier and cheaper.

#### 6.3.2. Recycling

Reported solvent reuse covers three to five cycles (Table S1), and the same criterion applies in all three treatment regimes. Every study judges a reuse cycle by the properties of the resulting fibre, whether residual gum content, quality grade, or comparison with conventional double boiling, and never by any property of the solvent. The solvent is therefore treated as a reusable medium, not as a reagent whose chemical state is monitored: publications confirm that degumming capacity survives, but report no composition of the spent liquor after a run. The reported cycle numbers are also lower bounds, since each value simply marks where the experiment stopped. A solvent that still meets performance requirements after five cycles is recorded as such, and no study has documented a failed cycle. The quoted number thus measures the extent of the experiment, not the operational limit. The three regimes handle spent liquor differently, by separating insoluble solids, by replenishing the active component, or by direct reuse without purification, yet all three are evaluated on the same basis, the quality of the resulting fibre.

## 7. Solvent Reuse, Product Valorisation and Downstream Integration

### 7.1. Recovery and Reuse of the Solvent

#### 7.1.1. The Scope of Recovery in This Field

Recovery and reuse are usually discussed together, as though one implied the other, and in this literature they are not the same thing. What the degumming studies do is limited: insoluble lignin and polysaccharides are separated out of the spent liquor [52], the liquor is topped up with fresh reagent when the active component has been consumed [51], or it is used again with no purification at all [50,59]. Separation by extraction, by anti-solvent precipitation as a designed step, or by membrane, does not appear in any of the eighteen studies collected here. The pattern is better described as continued use than as recovery. The studies also differ in what they count as a successful cycle: one requires that tenacity and residual gum still meet downstream requirements at the fifth cycle [47], while the others report only that performance was retained [50,51,59].

#### 7.1.2. Fate of the Dissolved Components

In all these studies the fibre is the analyte that is characterised. The spent liquor contains substances stripped from fibre, yet this liquid phase remains unmonitored. Three reporting gaps recur. Three fractions, namely lignin, hemicellulose and pectin, dissolve into the liquor during processing. As shown in Section 7.1.1, the adopted strategy for this liquor is recycling, either directly or after removal of insoluble solids. Separating insoluble matter does not equate to characterising dissolved components: no available work reports the composition of spent degumming liquor at any reuse cycle. Insights regarding accumulated substances are derived from research outside the scope of degumming. Zhang et al. [82] attributed the gradual decline in delignification efficiency across repeated cycles to hemicellulose, supporting the hypothesis of component accumulation. Nevertheless, their evidence originated from fractionation experiments where pectin was not investigated. Pectin is one major target of degumming, while its evolution during successive cycles remains unreported; hence this finding cannot be extrapolated to degumming systems. Alkaline systems follow a different rationale. Song et al. [51] replenished the liquor with neat sodium hydroxide, indicating reagent consumption instead of solvent degradation, though no comparison with fresh liquor was provided.

#### 7.1.3. Cost and Energy

Cost claims for DES degumming rest on a small number of estimates, and those estimates do not share a boundary. Haque et al. [57] estimated the cost of chemicals, energy and water over four cycles and found it better than the conventional route, the only cost estimate among the eighteen studies. Nie et al. [61] reported reductions of 48.9% in chemicals, 66.7% in water and 66.8% in time, but these are savings within a single combined process, not cycle-averaged costs. Liang et al. [83] gave 8.4 kWh per kg and 3.0% of purchase price for the recovery unit alone, excluding biomass handling and effluent disposal. Summing these datasets or citing them for mutual validation treats three partial records as a complete cost balance sheet. All three studies overlook the largest contributor: the solvent itself. Current costing accounts only for purchased raw materials and utility consumption. Solvent lost with washed fibre or diluted into wastewater, if included at all, is booked under effluent treatment expenses.

A single illustrative calculation shows the significance of this omission. The outcome is governed primarily by the bath ratio and fibre cost rather than solvent price, so a range bound is more appropriate than a single fixed value. Let f denote the price of degummed fibre, R the mass of solvent charged per unit mass of fibre, and p the delivered cost of the solvent. If reagents may account for no more than about one third of the operating cost, the allowable fractional solvent loss per cycle is L ≈ 0.3 f/(R p). At the 1:20 fibre-to-DES ratio used for hemp [55,57], a CU solvent at about US$1 per kilogram together with degummed fibre at US$2 to 5 per kilogram places L between roughly 3% and 15% of the solvent charged per cycle. The bound is loose enough to be met, which highlights the key issue: the target itself is not impractical, yet there are no measured data to evaluate whether it can be satisfied.

### 7.2. Separation and Valorisation of the By-Products

Degumming generates a spent liquor containing dissolved lignin, hemicellulose and pectin, yet the studies covered in this review barely explore this stream. Huang et al. [47] fractionated lignin out of the spent liquor and reported its purity, whereas Lin et al. [52] merely separated the insoluble lignin and polysaccharides during four reuse cycles. By-products are not a product stream in this field, so their valorisation is a possible extension of the work. The valorisation literature is well developed for lignin, covering catalytic hydrogenolysis [84], biological funnelling of heterogeneous aromatics [85], bacterial conversion to lipids, furfural, vanillin and polyhydroxybutyrate [86], and bioplastic and carbon fibre precursors [87], but it was built for feedstocks far richer in lignin than a bast fibre. Fernandes et al. [88] recovered 95 wt% of the lignin in maritime pine sawdust, a feedstock carrying on the order of a quarter of its mass as lignin, so the same separation route would meet a much smaller load per unit volume here. Ramie, the crop with the most developed degumming literature, is therefore also the weakest candidate for lignin valorisation, and whatever value that chain creates will have to come from the cellulose and from the hemicellulose and pectin fractions, not from lignin. Hemicellulose is in a worse position, since Zhang et al. [82] identified it as the cause of declining performance over recycling, which makes it a fraction the process must remove, not one it wants to recover. Basak et al. [89] showed what resolving that conflict would require, separating the fractions first and converting them afterwards, but on rice straw, not on a bast fibre. One route avoids the question altogether: Mouro et al. [90] dissolved hemp agro-waste in ChCl–glycerol and CU and electrospun the blend with poly(vinyl alcohol), so the solvent became part of the product instead of being taken back.

### 7.3. Integration with Downstream Processing

#### 7.3.1. Spinning: The Limit of the Evidence

Every degumming study in this review ends its evaluation at the refined fibre, and several close with a claim about downstream use. Fibres are reported to meet the Chinese national standard for refined ramie or to match the alkali route [48,50,51,62], and in one case the cellulose content, yield, fineness, tenacity, crystallinity and DP were all reported to meet spinning requirements [52]. These are statements about the fibre, not about yarn, and they support only the conclusion that the fibre is a usable spinning input. Yarn strength depends on the length distribution, the dispersion of fineness, the short-fibre content and the cohesion between fibres, and none of the studies here reports length distribution or short-fibre content in a form that would allow a yarn to be predicted. “Spinnable” is therefore treated in this review as a reasonable expectation drawn from fibre-level indices, not as a demonstrated result, and the same applies to the reports of a smooth, clean fibre surface [61,62], whose meaning differs between the textile and the composite context (Section 7.3.2).

#### 7.3.2. Composites and Moulded Pulp

In a composite, the surface that the degumming literature reads as evidence of completeness is not necessarily an advantage. The interfacial model proposed by Lee et al. [91] accounts for interdiffusion, electrostatic adhesion, chemical adhesion and mechanical interlocking. Within this framework, the physical and thermal properties and the flexural strength of plant-fibre composites are strongly governed by interfacial adhesion. Surface roughness and grooves create the mechanical interlocking effect. Nie et al. [61] and Song et al. [62] reported a smooth, clean surface as evidence that degumming was complete. Such a surface offers less interlocking and would have to be compensated by sizing, by coupling agents, or by a modification step of the kind surveyed by Ramachandran et al. [92] and Mohammadi et al. [93]. No study here has measured the interfacial strength, or the surface energy, of a DES-degummed bast fibre against any matrix.

One further pairing follows from the fibre architecture, and not from any reported result: foam forming prefers long fibres, which suits these crops, and moulded pulp is the incumbent it is measured against [94]. Neither has yet been tried on a DES-degummed fibre.

#### 7.3.3. Nanocellulose: One Solvent Family, Two Incompatible Duties

Acidic DES routes to cellulose nanofibrils are not pursued here, because nanocellulose production is premised on disrupting the aggregated state of cellulose, which is the opposite of the preservation criterion degumming is held to. The nanocellulose literature resolves the conflict by dividing the labour. The ChCl-based systems that are neutral act as swelling and pretreatment media and leave the disintegration to mechanical or enzymatic steps, for example, ethylene glycol under screw extrusion [95] or a DES followed by enzymatic hydrolysis [96]. The systems that disintegrate by themselves are the acidic ones, which hydrolyse amorphous cellulose and therefore sit on the far side of that line [97,98].

The practical implication for a degumming process is therefore a division of duty, not a new formulation: one solvent to swell and pretreat, a second step to disintegrate. One formulation cannot optimise both duties, and a fibre degummed by a preserving route has to be passed to a mechanical or enzymatic step, or to a different solvent, if nanocellulose is the target. The economics of such a sequence are unexamined. Almeida et al. [98] found the analysis absent, and Shen et al. [99], surveying nanocellulose from bast fibres, do not consider degumming residue as a feedstock.

## 8. Challenges and Outlook

### 8.1. Solvent Recovery and Regeneration: The Gap Between Description and Practice

The literature characterises DES as recoverable and regenerable, and regards this feature as the source of its environmental benefits. Nevertheless, bast fibre degumming has not attained such an operational state. Existing studies merely separate insoluble solids, replenish the liquor with fresh reagents, or reuse the spent liquor without purification. The widely cited 3–5 reuse cycles reported in this field span three distinct operational practices instead of representing a measured material property. Three quantities are consistently absent from the record: solvent loss per cycle, the resource requirement for regeneration, and the threshold at which liquor becomes unusable.

The gap matters because the solvent is the largest input to the process, by both mass and cost. A route that does not close its solvent loop has neither a cost base nor a demonstrable environmental claim, whatever the chemistry of the solvent. Closing that loop is the point at which a laboratory result has to become a process. The gap is difficult to close for reasons that belong to degumming, not to the solvents. The liquor is not clean and does not stay constant, because it carries the lignin, hemicellulose and pectin fragments released from the fibre together with the sugars and organic acids that accompany them. As these accumulate, the solvent’s own properties change, including water content, viscosity and the availability of the hydrogen bonds that do the work, so a liquor in its third cycle is no longer the same solvent as in its first, and no study reports which property fails first. The solvent also does not leave the process only in the liquor: the fibre has to be washed, and washing disperses the solvent into a large aqueous stream whose treatment is a different operation from the regeneration of a concentrated liquor. Most of the loss is likely to occur in that wash, and no degumming study quantifies it.

Regeneration must be tailored to crop-specific spent liquor, because the components requiring removal are determined by substances leached from the raw crop. The pectin-rich gum of ramie and lignin-rich gum of hemp impose distinct separation challenges. A regeneration strategy validated for one crop is therefore not directly applicable to others without re-optimisation. Furthermore, the success criterion is not purely chemical but also textile-related: the reusability of recovered solvent hinges on the quality of the resulting fibre. Accordingly, the performance target for regeneration is defined by downstream outcomes after the regeneration operation. Theoretical accounts focus on neat solvent, whereas the practical system uses solvent after treatment of a specific crop and dilution by wash water. This discrepancy is the practical bottleneck for scaling up this chemistry to industrial plant operation.

### 8.2. Evaluating a Degumming Result: Several Dimensions, and No Weights

Degumming outcomes are characterised across multiple dimensions simultaneously: gum removal, fibre yield, fineness, staple length, breaking tenacity, elongation, crystallinity, DP, and surface morphology. These performance parameters do not vary in tandem. In ramie, the system that left the least gum, ethylamine hydrochloride–ethylene glycol at 3.15%, gave the weakest fibre of those compared, at 3.05 cN/dtex, while the system that left more gum, CU at 3.76%, gave the strongest, at 6.51 cN/dtex [47]. In hemp, a route that removed only 53% of the non-cellulosic components produced fibre with a breaking tenacity 25% above alkali-degummed fibre, and with greater length and a higher DP [54]. A result of this kind cannot be reduced to a single index, so this review reports gum removal and fibre properties in separate columns and computes no aggregate score.

Ranking two different processes therefore requires weighted evaluation criteria, yet no such weighting scheme is available from the existing literature. Studies performing optimisation based on a composite assessment of yield, residual gum, fineness and tenacity report only their selected operating point, never the maximum of a predefined objective function. Consequently, the obtained optimum cannot be reproduced in other settings, even when using the identical raw material. The same indeterminacy governs the evidence for the mechanism, where most studies stop at methods that cannot identify a bond, so the sequence of events is asserted more finely than it is measured (Section 6.1.3). The difficulty is not the individual measurement, because the fibre tests are standardised and single-valued and the standards available define one property each (ASTM D3822, ISO 1973). What is missing is the rule that combines them. Neither the indicator set nor its weighting has been fixed for degumming by any standard, round-robin or consensus protocol, and that is an objective obstacle to comparison rather than a shortcoming of particular studies.

From that gap two specific changes follow, and neither needs new equipment. The first is to state the weighting, or at least the ordering. A study that reports its optimum as a defined criterion, or that simply declares tenacity more important than residual gum, leaves a result others can reproduce and contest. The same numbers without that rule cannot be compared at all. The second requirement is a minimum reporting standard to unify the format of results across separate studies. The checklist defines mandatory reporting items. For raw feedstock, researchers should record cultivar, stem fraction, and the processing stage for compositional analysis. For fibres, reports need residual gum content, component removal rates relative to a defined reference, fineness (with measurement unit and test method), and breaking tenacity together with test conditions, gauge length and test standard. For treatment processes, crystallinity and DP (with test methods), as well as reusability data, including regeneration protocol and cycle termination criteria, should be provided. Rafi et al. [18], reviewing DES degumming for hemp independently, reach the same conclusion about the absence of standard protocols. The obstacle lies in convention, not in capability; this review therefore treats the reporting problem as solvable.

## 9. Conclusions

This review set out to place the five crops within a common comparative framework, which alters the picture presented by the literature on any individual crop. The field has converged on a narrow family of solvents. ChCl is the hydrogen bond acceptor in nearly every system, and CU is regarded as the best system on ramie and kenaf and the most balanced on hemp. Imidazole, ethylene glycol, lactic acid, ETA and monoethanolamine extend the donor range without displacing it. Operating temperatures fall between 100 and 175 °C, and microwave assistance compresses the treatment into minutes. Residual gum has been brought to 3.15% on ramie, by a system that gave the weakest fibre of the three tested, and to 9.42% on kenaf, in both cases against the double-boiling route used as the benchmark. Crystallinity rises, at the extremes from 65.7% to 83.3%. Reuse has been demonstrated for three to five cycles under three different regimes, the longest of these without any purification.

In short, the underlying chemistry is more mature than the corresponding process technology. DES degumming has been proven feasible for all five bast fibre crops, yet none have undergone scaled validation. The missing element is not an improved solvent, but quantitative datasets together with frameworks for their interpretation: a solvent mass balance to enable process cost estimation, weighting criteria to permit comparison between different degumming outcomes, and evaluation methods to quantify the claimed environmental benefits.

The cross-crop comparison also narrows what may be claimed. Part of the agreement visible across the five crops reflects a shared experimental lineage, not independent confirmation, so the convergence of the field is smaller than the number of its papers suggests. What the comparison does establish is where the difficulty lies. The solvent chemistry of these systems is understood at a depth that no other bast fibre degumming route has approached, covering bond cleavage, unit selectivity and competition for hydrogen bonds; yet that understanding rests on two crops, two laboratories, and fibres never tested beyond the fibre itself. A defensible claim about DES degumming therefore calls for a series of extended investigations, not another solvent.

## Figures and Tables

**Table 1:** The five classes of DES and their relevance to bast fibre degumming ^1^.

Type	HBA	HBD	Representative Example	Relevance to Fibre Degumming
I	Quaternary ammonium salt	Metal chloride	ChCl + ZnCl_2_	Metal-based and not used on textile fibres
II	Quaternary ammonium salt	Hydrated metal chloride	ChCl + CoCl_2_·6H_2_O	Metal-based and not used on textile fibres
III	Quaternary ammonium salt	Amide, carboxylic acid or alcohol	ChCl + urea, ChCl + oxalic acid, ChCl + glycerol, ChCl + ethanolamine	The systems on which essentially all reported degumming work relies
IV	Metal chloride hydrate	Amide or alcohol	ZnCl_2_ + urea	Occasional use in biomass fractionation, but not in degumming
V	Non-ionic molecular species	Non-ionic molecular species	Thymol + menthol, lidocaine + fatty acid	Hydrophobic, mainly used for extraction and of limited value in aqueous fibre processing

^1^ Adapted from the type I to IV classification consolidated by Smith et al. [36]. The relevance column is specific to the degumming literature surveyed in this review. Abbreviations: HBA (hydrogen bond acceptor), HBD (hydrogen bond donor), ChCl (choline chloride) and ethanolamine (ETA).

## Data Availability

No new data were created or analysed in this study. Data sharing is not applicable to this article.

## Supplementary Materials

The following supporting information can be downloaded at https://www.mdpi.com/article/10.3390/molecules31213911/s1, Table S1: Cross-crop comparison of the DES systems used for bast fibre degumming.

## Abbreviations

The following abbreviations are used in this manuscript:

2D-NMR	Two-dimensional nuclear magnetic resonance
ChCl	Choline chloride
CI	Choline chloride–imidazole
CU	Choline chloride–urea
DAHD	The integrated DES–alkaline hydrogen peroxide degumming route (as named by Ma et al.)
DES	Deep eutectic solvent(s)
DP	Degree of polymerisation
ETA	Ethanolamine
HBA	Hydrogen bond acceptor
HBD	Hydrogen bond donor
Py-GC/MS	Pyrolysis–gas chromatography/mass spectrometry
FTIR	Fourier transform infrared spectroscopy
SEM	Scanning electron microscopy
XRD	X-ray diffraction
DFT	Density functional theory
