# Supplementary material for: Deep Eutectic Solvents for Bast Fibre Degumming: A Cross-Crop Review of Systems and Mechanisms
Source: Molecules. 2026 Oct 29;31(21):3911. doi: 10.3390/molecules31213911 (PMC13764869; doi:10.3390/molecules31213911)
Supplement: Supplementary file 1 [file molecules-31-03911-s001.zip › molecules-4617691-supplementary.pdf]

Table S1. Cross-crop comparison of the DES systems used for bast fiber degumming.

Crop	Study	DES system[1]	Conditions	Degumming outcome	fiber properties	Solvent reuse	Mechanistic and characterisation evidence
Ramie	Song et al. [48]	DES applied after a steam-explosion pretreatment	Steam-explosion pretreatment followed by DES treatment; alkali-free route	Degumming achieved with no alkali; fiber quality comparable to the alkali route	Residual gum 4.68%; average fineness1420 Nm; breaking tenacity 5.98 cN/dtex; all meeting the Chinese national standard	Not reported	FTIR, SEM and XRD confirming degumming
Ramie	Huang et al. [47]	Three systems compared: CU (choline chloride–urea), CI (ChCl–imidazole) and ethylamine hydrochloride–ethylene glycol	Single-step boil for 2 h, with no auxiliary treatment	CU left 3.76% residual gum, close to the alkali route; EE was lowest at 3.15%; all three removed most non-cellulosic components	CU 6.51 cN/dtex; CI highest elongation at break (3.30%) with 5.50 cN/dtex; EE weakest at 3.05 cN/dtex	Reusable repeatedly; after the fifth cycle both tenacity and residual gum still met downstream requirements	Lignin recovered in fractions from the spent liquor (purity 9.54%)
Ramie	Huang et al. [49]	The CU, CI and ethylamine hydrochloride–ethylene glycol systems with 0.2 g/L anthraquinone (AQ) as cellulose protectant	Same conditions as the parent systems, with no auxiliary treatment	AQ accelerates lignin removal while protecting the carbohydrates from excessive degradation	DP +3.2%, crystallinity +5.56%, breaking tenacity +8.44%, elongation at break +11.9%, work of rupture +33.3%; degradation temperature raised	Not reported	Comparison with the same DES without the protectant (chemical structure and thermal properties)
Ramie	Lin et al. [52]	ChCl–urea	Process parameters optimised by orthogonal design	Most non-cellulosic components still removed after four successive degumming runs	Cellulose content >90.2%; fiber yield 74.2–78.6%; fineness 6.30–7.87 dtex; tenacity 5.21–6.72 cN/dtex; crystallinity 65.7–70.1%;	Four cycles, with insoluble lignin and polysaccharides separated	SEM, chemical composition, XRD, TGA, DP

					decomposition temperature 353–356 °C; DP 1679–2445; all meeting spinning requirements		
Ramie	Song et al. [50]	ChCl–monoethanolamine (ChCl:MEA) 1:8, an alkaline DES	140 °C, selected by a combined assessment of yield, residual gum, fineness and breaking tenacity	Very low hemicellulose and lignin residues; cellulose content above that of conventional alkali boiling	Crystallinity and thermal stability improved and comparable to conventional alkali treatment; refined fiber reaching the third grade of the Chinese national standard	Three successive cycles without purification, with degumming performance retained	SEM, FTIR, XRD, TGA
Ramie	Song et al. [51]	ChCl–ethylene glycol 1:6 with 0.6% NaOH (alkali-promoted)	140 °C, selected on residual gum	Lignin and hemicellulose substantially removed	Single-fiber crystallinity and temperature of maximum degradation rate raised; macroscopic physical and mechanical properties meeting the national standard for refined ramie	Spent liquor topped up with fresh NaOH and reused, with degumming capacity retained	SEM, FTIR, XRD, TGA
Hemp	Ahmed et al. [55]	DES combined with microwave energy (MWE)	fiber-to-DES liquor ratio 1:10 and 1:20	Pectin, oil, wax and other gum, lignin and amorphous cellulose removed; holocellulose 98.63% against 98.87% for the alkali route	Thermal stability improved (enthalpy 171.34 J/g against 272.09 J/g for the raw fiber); UPF 118.11 (1:10) and114 (1:20), meeting UV-protective textile requirements	Not reported	SEM, chemical composition, FTIR, XRD, thermal analysis
Hemp	Ahmed et al. [56]	Several DES systems combined with microwave energy (MWE)	Microwave–DES combination, with a very short treatment time	Non-cellulosic components and impurities removed synergistically without damaging the cellulose structure; residual lignin 2.88%	Crystallinity up to 83.27% (ChCl–urea system); maximum degradation temperature 356 °C; bacterial growth inhibited	Not reported	Systematic characterisation by advanced analytical techniques (SEM, FTIR, XRD, thermal analysis)

Hemp	Ma et al. [54]	ChCl–urea 1:2 combined with alkaline H_2O_2 (the DAHD route)	Temperature, time and liquor ratio examined systematically	53% removal of non-cellulosic components; cellulose content above that of the alkali route	Breaking tenacity +25%, length +30% and DP +20% against conventional alkali	Adequate degumming performance after reuse	Py-GC/MS (levoglucosan from cellulose +27.8%; esters −1.07%, ketones −11.1%); 2D-NMR (β-O-4′ linkages substantially cleaved, β-5′ eliminated, G-type lignin degraded more than S-type)
Hemp	Mouro et al. [90]	ChCl:glycerol 1:2 and ChCl:urea 1:2 (dissolution of hemp agro-waste)	Electrospinning after blending with PVA	The waste fraction is converted directly into functional nanofibers, with no separate fiber-separation step	fiber diameter 258 ± 65.6 to 381 ± 134 nm; tensile strength and Young's modulus increased; antioxidant and antibacterial activity	Not reported	SEM and related characterisation
Hemp	Gao et al. [53]	CU, ethylenediamine hydrochloride–ethylene glycol and CI (ChCl–imidazole)	CI 3:8 at 175 °C for 2.5 h; EE 1:2 at 175 °C for 3 h	Pectin, hemicellulose and lignin removed from the amorphous regions; cellulose share rising from 54% to about 80%	None of the three damages the cellulose structure or crystalline form; CI is best for hemicellulose and pectin, EE for lignin, and CU is the cheapest and the most balanced of the three (proposed as the industrial choice)	Not reported	DFT calculations supporting the configurational stability of the three systems
Hemp	Firouzi et al. [58]	ChCl–glycerol, selected from three systems	RSM (central composite design) over time, temperature, DES-to-hemp ratio and glycerol:ChCl	Cellulose:lignin ≈ 70.8:17 at the optimum (reported as 70.817)	Crystallinity index 81.4%; cellulose yield58.1% (untreated) → 69.7% (sample h-12)	Not reported	SEM, FTIR, TGA, XRD with machine-learning prediction (Random Tree best, R^2 =0.855)

			molar ratio				
Hemp	Haque et al. [57]	Ternary DES: ChCl/lactic acid/glycerol (1:5:2) and ChCl/lactic acid/urea (1:5:2), against binary ChCl/lactic acid	105 °C, 2 h, liquor ratio 1:20	Non-cellulosic components effectively removed, with better fiber separation	Crystallinity increased; tenacity better than the conventional route and in less time; structural and thermal analysis confirming that cellulose integrity was preserved	≥4 cycles; cost estimate over four cycles (chemicals, energy, water) better than the conventional route	Structural and thermal analysis
Hemp	Rafi et al. [18], review	Review of ChCl–urea and related DES systems	Moderate temperatures	>90% cellulose retained with near-complete lignin removal; notes that hemp lignin is higher than ramie and that semi-degumming is required to preserve fiber integrity	—	Recyclability and durability emphasised; hybrid processes reported to cut treatment time by 50%; gaps identified in cost-effectiveness and standard protocols	Critical review
Kenaf	Nie et al. [61]	DES combined with microwave and alkaline ultrasonic treatment	Combined process	Gum removed effectively, giving a smooth clean fiber surface; residual gum 9.42%, comparable to the conventional two-step alkali boil	Crystallinity index66.7%; fineness 4.13 tex; breaking tenacity 13.7 cN/tex	Chemicals, water and time reduced by 48.9%, 66.7% and 66.8%	Chemical composition, surface structure, thermal properties
Kenaf	Nie et al. [60]	CU, selected from a screen of one HBA with three HBDs	160 °C, 2 h	CU, described by its authors as an alkaline amide-type DES, gave the best degumming, removing gum effectively and yielding refined kenaf fiber	Morphology, chemical composition, crystallinity, DP and physical properties systematically evaluated	At least three cycles	Cl^- competition weakening intra-fiber hydrogen bonds → phenolic hydroxyl deprotonation with β-O-4 cleavage, removing different lignin units

Kenaf	Song et al. [59]	ChCl–ethanolamine (ChCl–ETA) 1:6, an alkaline DES	120 °C, selected on residual gum	Residual gum comparable to the conventional double-boiling route	Characterised in full (chemical composition, SEM, FTIR, XRD, TGA, 2D-NMR)	≥5 cycles without purification, still comparable to double boiling	2D-NMR linkage analysis
Flax	Xiang et al. [46]	ChCl–urea combined with a microbial step	Orthogonal optimisation: 100 °C, 120 min, 90% DES	Pectin 86.4%, hemicellulose 26.7% and lignin 55.1% removed	Breaking tenacity 16.77 cN/tex, better than the microbial step alone	NMR confirming that the DES composition was unchanged; the whole batch of liquor reusable	Chemical analysis, SEM, FTIR, XRD, TGA, NMR
A. venetum	Song et al. [62]	ChCl–urea 1:2 combined with alkali	Microwave-assisted DES pretreatment, optimised at 110 °C for 60 min	The combined DES–alkali route removed hemicellulose and lignin effectively, giving a smooth clean surface	Crystallinity increased and average cellulose molecular weight decreased (DP↓), with thermal stability markedly improved; residual gum, length, fineness and breaking tenacity of the refined fiber comparable to the alkali route	Not reported	Chemical composition, surface microstructure, crystallinity, DP, thermal properties

1 Abbreviations used for the solvent systems: ChCl (choline chloride), CU (ChCl–urea), CI (ChCl–imidazole), MEA (monoethanolamine) and ETA (ethanolamine). DAHD is the label the authors give to their own combined DES and alkaline hydrogen peroxide route.
